# Does neutrophil to lymphocyte ratio demonstrate deterioration in renal function?

**DOI:** 10.1080/0886022X.2018.1455590

**Published:** 2018-04-04

**Authors:** Senol Tonyali, Cavit Ceylan, Sedat Yahsi, Mine Sebnem Karakan

**Affiliations:** a Clinic of Urology, Turkiye Yuksek Ihtisas Training and Research Hospital, Ankara, Turkey;; b Clinic of Nephrology, Turkiye Yuksek Ihtisas Training and Research Hospital, Ankara, Turkey

**Keywords:** Neutrophil, lymphocyte, nephrectomy, chronic kidney disease, renal failure

## Abstract

**Introduction:** Chronic kidney disease (CKD) is a major health issue worldwide, which leads to end-stage renal failure and cardiovascular events. Neutrophil to lymphocyte ratio (NLR) is a surrogate marker of inflammation and has been widely studied in malignancies, hypertension, heart diseases, and vascular diseases. In this study, we aimed to investigate if NLR represents renal reserve and function after partial or radical nephrectomy.

**Methods:** We conducted a retrospective study consists of patients who had undergone radical/partial nephrectomy in our hospital and/or who admitted to urology and nephrology clinics as an outpatient. Patients were divided into four groups: Group 1 (*n* =  46): Healthy controls; Group 2 (*n* =  50): Patients who had undergone unilateral partial nephrectomy; Group 3 (*n* =  46): Patients who had gone unilateral nephrectomy; Group 4 (*n* =  82): Patients who had CKD.

**Results:** The mean NLR of each group was as follows: Group 1: 2.14 ± 0.73; Group 2: 3.52 ± 3.74; Group 3: 3.64 ± 3.52, and Group 4: 3.53 ± 2.30. NLR was lower in Group 1 compared to other groups but statistically significant difference was observed only between Group 1 (control) and Group 4 (CKD), 2.14 ± 0.73 versus 3.53 ± 2.30 (*p* = .005). In non-parametric correlation analysis NLR was found negatively correlated with GFR and positively correlated CKD stage (*p* = .028 for both correlations).

**Conclusions:** The NLR may constitute a practical predictor of CKD besides Cr in patients who had undergone partial or radical nephrectomy.

## Introduction

Chronic kidney disease (CKD) is a major health issue worldwide, which leads to end-stage renal failure and cardiovascular events. There is a steady increase in the number of patients with CKD. The prevalence of CKD in Turkey was 15.7% in a population-based study conducted with 8765 patients. And ∼62,000 patients are on replacement therapy [1].

The role of inflammation in acute kidney injury [2] and end-stage kidney disease (ESKD) is well-recognized [3].

Neutrophil–lymphocyte ratio (NLR) is a surrogate marker of inflammation and has been widely studied in malignancies [4–6], hypertension, heart diseases and vascular diseases [7,8].

In this study, we aimed to investigate if NLR represents renal reserve and function after partial or radical nephrectomy.

## Methods

After the institutional review board approval, we conducted a retrospective study consists of patients who had undergone radical/partial nephrectomy in our hospital and/or who admitted to urology and nephrology clinics as an outpatient. Patients were divided into four groups: Group 1 (*n* = 46): Healthy controls; Group 2 (*n* = 50): Patients who had undergone unilateral partial nephrectomy; Group 3 (*n* = 46): Patients who had gone unilateral nephrectomy; Group 4 (*n* = 82): Patients who had CKD. Neutrophil/Lymphocyte ratio was calculated by means of complete blood count (CBC) values. CBC results at least 1 month after surgery were used for nephrectomy patients. Glomerular filtration rate (GFR) was calculated using Modification of Diet in Renal Disease (MDRD) equation in CKD patients and the patients were divided into five groups according to GFR. Active urinary tract infection was eliminated in all controls by means of urine analyzes and detailed anamnesis. Patients who were still on renal replacement therapy were also excluded from the study.

Mean ± standard deviation (SD), minimum and maximum values were used to describe the quantitative variables. Normality assumption was checked by Shapiro–Wilk’s test and it was found that data do not conform normal distribution. Nominal data were compared using the Chi-square test. Threshold was calculated via ROC curves and Youden Index. Comparison of quantitative measurements among patients and controls were assessed with the non-parametric Spearman correlation analysis. Comparison between groups made by using Mann–Whitney *U* and Kruskal–Wallis tests. Bivariate and multiple regression analyzes were used to analyze factors associated with CKD. predict For all analyzes the IBM-SPSS version 21.0 was used and the statistical significance was set at *p* < .05.

## Results

In total 224 patients were included the study whom 149 (66.5%) were males and 75 (33.5%) were females. There were 46, 50, 46 and 82 subjects in Group 1, Group 2, Group 3 and Group 4, respectively. The distribution of male and females did not differ between the four groups (*p* = .341). The mean age of the patients was 60 ± 11.2 years. There was no significant difference between Group 1, Group 2, and Group 3 in terms of age (*p* = 1.0) however the mean age of patients in Group 4 was higher than the mean age of patients Group 1 and Group 2 (*p* = .00, *p* = .003, respectively). The mean NLR was 3.2 ± 2.8 and the Cr was 1.5 ± 0.8 mg/dL in the entire cohort. The mean NLR of each group was as follows: Group 1: 2.14 ± 0.73 (median: 2.0); Group 2: 3.52 ± 3.74 (median: 2.1); Group 3: 3.64 ± 3.52 (median: 2.3), and Group 4: 3.53 ± 2.30 (median 2.8). The mean NLR in control group did not differ between males and females, 2.1 ± 0.8 and 2.2 ± 0.5, respectively (*p* > .05). The mean eGFR value was lowest in Group 4 and highest in Group 2 (Group 1:77.6 ± 17.3 mL/min/1.73 m^2^, Group 2: 80.1 ± 19.7 mL/min/1.73 m^2^, Group 3: 53.5 ± 15.4 mL/min/1.73 m^2^, Group 4: 30.7 ± 10.6 mL/min/1.73 m^2^. NLR was lower in Group 1 compared to other groups but statistically significant difference was observed only between Group 1 (control) and Group 4 (CKD), 2.14 ± 0.73 vs. 3.53 ± 2.30 (*p* = .000) (Table 1).

**Table 1.** Patient demographics and characteristics.

	Group 1 (*n* = 46)	Group 2 (*n* = 50)	Group 3 (*n* = 46)	Group 4 (*n* = 82)	*p* value
Age (years)	56.1 ± 10.3	57.2 ± 10.1	59.3 ± 11.2	64.1 ± 11.2	.000
Sex					
Male	32	28	31	58	.341
Female	14	22	15	24	
GFR (mL/min/1.73 m^2^)	77.6 ± 17.3	80.1 ± 19.7	53.5 ± 15.4	30.7 ± 10.6	.00
NLR	2.14 ± 0.7 (2)	3.52 ± 3.7 (2.1)	3.64 ± 3.5 (2.3)	3.53 ± 2.3 (2.8)	
NLR < 3.18	42 (91.3%)	38 (76%)	31 (67.4%)	46 (56.1%)	.000
NLR > 3.18	4 (8.7%)	12 (24%)	15 (32.4%)	36 (43.9%)	

In multiple regression analysis NLR and Cr together was found related with decreased functional nephron reserve such that estimated nephron reserve decreases while group number increases (Table 2).

**Table 2.** Effects of Group, Group–Creatinine, Group–GFR and Group?Creatinine-GFR on NLR (regression analysis).

	*β*	SE for *β*	*p*	% change for *β*
Group	0.109	0.035	.002	
Group	0.062	0.041	.130	43.12
Creatinine	0.117	0.055	.034	
Group	0.087	0.043	.044	20.13
GFR	–0.002	0.002	.370	
Group	0.074	0.043	.087	32.11
Creatinine	0.167	0.079	.034	
GFR	0.002	0.003	.374	

The cutoff value for NLR associated with CKD (GFR< 60 mL/min/1.73 m^2^) was 3.18, with 39% sensitivity and 81% specificity (*p* = .11). 67 patients had a NLR value >3.18 and 157 patients had a NLR value <3.18. To have a NLR value >3.18 was associated with 2.8-fold increased risk to have CKD [95% CI (lower: 1.5; upper: 5.2)].

In non-parametric correlation analysis NLR was found negatively correlated with GFR and positively correlated CKD stage (*p* = .028 for both correlations; Table 3).

**Table 3.** The correlation analysis of NLR, GFR and CKD stage.

	NLR	GFR	CKD stage
Spearman’s rho			
NLR			
Correlation coefficient	1.000	−.164^a^	.164^a^
Sig. (two-tailed)		.028	.028
*N*	178	178	178
GFR			
Correlation coefficient	−.164^a^	1.000	−.943^b^
Sig.(two-tailed)	.028		.000
*N*	178	178	178
CKD stage			
Correlation coefficient	.164^a^	−.943^b^	1.000
Sig.(two-tailed)	.028	.000	
*N*	178	178	178

a Correlation is significant at the 0.05 level (two-tailed).

b Correlation is significant at the 0.01 level (two-tailed).

## Discussion

NLR is a widely utilized biomarker of systemic inflammation, which can be obtained easily from CBC. Many studies have been conducted to demonstrate the value of NLR in various conditions such as hypertension [7], cardiac disorders [8], malignancies [4–6,9–11], and renal failure [3,12–14].

Inflammation has proven to be associated with CKD and CKD patients has shown to posses a low-grade inflammatory status [15]. NLR was found increased in patients not only receiving hemodialysis but also in predialysis term [13,15,16]. And also improvement of inflammation status according to NLR was better in preemptive renal transplant patients compared to non-preemptive patients at the end of the first year post-renal transplantation [16]. Some researchers have reported that NLR and platelet–lymphocyte ratio (PLR) are positive correlated with inflammatory markers in patients with ESKD [14,17,18]. Ahbap et al. reported that patients with higher CRP (C-reactive protein) levels >3 mg/dL had higher NLR and PLR levels compared to patients with lower CRP levels [NLR (3.7 ± 0.2 vs. 2.7 ± 0.2, *p* < .01) and PLR (150.7 ± 6.9 vs. 111.8 ± 7.0, *p* < .001)] [17]. In a study by Turkmen et al., NLR >3.5 was found associated with increased level of TNF-α but not with CRP and IL-6 in patients with ESKD [14]. In another study, PLR was positively correlated with NLR, IL-6, and TNF (*p* < .001, *p* = .03, *p* = .008, respectively) and the authors concluded that PLR was superior to NLR in terms of inflammation in patients with ESKD [18].

NLR was also found associated with cardiovascular and all-cause mortality in hemodialysis patient (*p* = .0040 and *p* = .0021, respectively) [3]. Although there has been numerous studied in the literature regarding to association between inflammation and NLR in ESKD, there is not any about the possible relation between renal reserve and NLR. In the present study, we found NLR to be significantly higher in patients with CKD compared to controls. And also NLR was found associated with the stage of CKD.

Renal cell carcinoma accounts for 2–3% of all cancers. RCC is the most common solid lesion within the kidney and represents 90% of all renal malignancies [19]. While radical nephrectomy (RN) is indicated for T2 tumors (tumor >7 cm), nephron-sparing surgery is the standard treatment for localized T1a-b tumors (tumors <7 cm) [19]. Because when achieving RCC free status, preserving of the renal function must be the core topic of the process. In previous studies, NSS was reported to be associated with preserved renal function compared to RN [20,21].

In the present study, we found NLR to be associated with GFR. This is the first study in the literature reporting the predictive value NLR on GFR in nephrectomy patients. In this manner, it might be possible to predict renal reserve and function by means of NLR after partial or radical nephrectomy.

Our study is not without limitations. First of all, this is a retrospective study. Given the retrospective nature of the study, it was not possible to obtain other inflammatory markers such as CRP, TNF-α and IL-6. And also the sample size of patients in each CKD stage group was small which weakened the power of some statistical analysis.

## Conclusions

The NLR may constitute a practical predictor of CKD besides Cr in patients who had undergone partial or radical nephrectomy. This is the first study in the literature demonstrating the predictive value NLR in proceeding to CKD. Larger, prospective studies including the long-term NLR, other inflammatory markers and renal functions values of nephrectomy patients are mandatory to establish definitive criteria.
